# Determinants of Benzodiazepine-Dispensing Practice Among Community Pharmacy Dispensers in Dar es Salaam, Tanzania

**DOI:** 10.1155/adpp/1404995

**Published:** 2025-06-03

**Authors:** Adhra R. Mansour, Joseph Matobo Thobias, Emili Yondu, Erick G. Philipo, Wigilya P. Mikomangwa, Manase Kilonzi, Alphonce Ignace Marealle, Ritah F. Mutagonda

**Affiliations:** ^1^Department of Clinical Pharmacy and Pharmacology, Muhimbili University of Health and Allied Sciences, Dar es Salaam, Tanzania; ^2^School of Public Health and Social Sciences, Muhimbili University of Health and Allied Sciences, Dar es Salaam, Tanzania; ^3^Department of Medicinal Chemistry, Muhimbili University of Health and Allied Sciences, Dar es Salaam, Tanzania; ^4^Department of Pharmaceutics and Pharmacy Practice, Muhimbili University of Health and Allied Sciences, Dar es Salaam, Tanzania

**Keywords:** benzodiazepine, community pharmacy, dispensers, dispensing practice, Tanzania

## Abstract

**Purpose:** This cross-sectional study assessed determinants of benzodiazepine (BZP)-dispensing practices among community pharmacy dispensers in Dar es Salaam, Tanzania.

**Methods:** A cross-sectional study involving 378 community pharmacy dispensers was conducted between March and June 2024. An adapted structured questionnaire was used to gather information on the sociodemographics, most dispensed BZPs, dispensers' knowledge, and dispensing practice of BZPs. Determinants of dispensing practice were determined by multivariable logistic regression analysis using SPSS Version 23.

**Results:** Of 378 dispensers, 232 (61.4%) were female, 263 (69.6%) had a college education level, and 193 (51.1%) were pharmaceutical technicians. Diazepam was the most dispensed BZP (163 (43%)), followed by lorazepam (102 (27%)). More than half, 203 (53.7%), of the dispensers had inadequate knowledge, and 240 (63.5%) of dispensers had good dispensing practices. Nonpharmaceutical dispensers were less likely to have good dispensing practice (AOR = 0.16, 95% CI (0.05–0.49)) whereas having adequate knowledge of BZPs (AOR = 2.64, 95% CI (1.64–4.25)) were significantly associated with the good dispensing practice of BZPs.

**Conclusion:** Knowledge levels and the type of pharmaceutical professionals are determinants in ensuring proper BZP-dispensing practices. These indicate the need for continuous professional development and stricter enforcement of dispensing regulations to improve pharmacy practices and prevent unauthorized BZP dispensing.

**Summary**

• Diazepam (43%) and lorazepam (27%) were the most commonly dispensed benzodiazepines (BZPs).
• More than 50% of community pharmacy dispensers had inadequate knowledge of BZP while 63.5% demonstrated good dispensing practices.
• Nonpharmaceutical dispensers exhibited poor dispensing practices whereas those with adequate knowledge were more likely to follow proper dispensing protocols.
• Literature from developing countries indicates that BZPs are among the most commonly abused medications. Furthermore, studies show that many community pharmacy dispensers in these regions are willing to dispense BZPs without a prescription. To develop appropriate interventions, it is essential to assess dispensers' knowledge, dispensing practices, and the factors influencing these practices.
• Between March and June 2024, we conducted a cross-sectional study in Dar es Salaam, Tanzania, to evaluate the knowledge, dispensing practices, and determinants of BZP dispensing among community pharmacy dispensers. Our findings revealed that diazepam and lorazepam were the most frequently dispensed BZPs. In addition, over 50% of dispensers had inadequate knowledge, while 63.5% demonstrated good dispensing practices. Nonpharmaceutical dispensers exhibited poor dispensing practices whereas those with adequate knowledge were more likely to follow proper dispensing protocols.
• These findings highlight the need for continuous professional development and stricter enforcement of dispensing regulations to improve pharmacy practices and prevent unauthorized BZP dispensing.

## 1. Introduction

Benzodiazepines (BZPs) are known for their diverse clinical effects on the central nervous system [1, 2]. For instance, BZPs cause varying degrees of hypnosis, amnesia, sedation, muscle relaxation, and actions against convulsions [2, 3]. Thus, BZPs are most prescribed and widely used globally [1, 3–6] in the field of psychiatry to treat insomnia, general anxiety disorder, panic attack, phobia, obsessive–compulsive disorder, posttraumatic stress disorder, treatment of muscle stiffness, and treatment neurological conditions like epilepsy [6–10].

Globally, estimates indicate that BZP use continues to increase due to the simplicity of their use, efficacy, availability, affordability, and long-acting duration [11–15]. Studies conducted in Australia, Canada, Spain, the USA, and France found that the use of BZPs increased with age from 14.7% in young adults to 12%–40% in older adults [1, 16–18]. A study conducted in South Africa reported 13.3%–49.5% use of BZP [13], and in Tanzania, abuse of BZPs by adolescents is reported to range between 5% and 12% [19, 20].

Despite BZPs having many clinical advantages, there are significant disadvantages associated with their misuse, which include memory impairment, asthenia, depression, emotional blunting, paradoxical pleasure, falls, sedation, high risk of abuse, dependence, and the potential to worsen or precipitate anxiety, irritability, and sleep disturbance if not dosed appropriately or discontinued abruptly [21–25]. For all these reasons, BZPs are classified as controlled drugs, and strict regulations and guidelines have been developed for their prescription and dispensing in hospital settings and community pharmacies [26–28].

Community pharmacies serve as primary healthcare (PHC) facilities in many low- and middle-income countries (LMICs) due to their accessibility and affordability [29–32]. As a result, they play a crucial role in promoting the rational use of medications. This is particularly important given the high prevalence of self-medication reported in LMICs [33, 34]. However, studies have highlighted poor dispensing practices of BZPs among community pharmacy dispensers [12, 20, 35]. For example, a 2019 survey in Tanzania found that 70% of community pharmacy dispensers were willing to dispense diazepam without a prescription [20]. Therefore, there is a need to assess the determinants of BZP-dispensing practice among community pharmacy dispensers. This study assessed determinants of BZP-dispensing practice among community pharmacy dispensers in Dar es Salaam, Tanzania.

## 2. Methods

### 2.1. Study Design

This community-based cross-sectional study was conducted in the Dar es Salaam region between March and June 2024.

### 2.2. Study Setting

The study was conducted in the Dar es Salaam region, the largest business center in Tanzania, with a well-established community pharmacy sector. According to the 2022 Tanzania census, the population of the Dar es Salaam region is 5,383,728 [36]. Dar es Salaam region comprises five administrative municipalities (Kinondoni, Ilala, Ubungo, Temeke, and Kigamboni) [36]. Community pharmacy business in Tanzania is technically regulated by the Pharmacy Council of Tanzania and Tanzania Medicines and Medical Devices Authority (TMDA). Currently, there are about 872 registered community pharmacies in the Dar es Salaam region [37].

### 2.3. Study Population, Sample Size, and Sampling Technique

The study involved community pharmacy dispensers working in registered pharmacies in Dar es Salaam, Tanzania. The sample size was estimated using the formula for cross-sectional surveys: *n*=(*Z*2*P*(100 − *P*))/*ε*2. Given the lack of similar studies in the East Africa region, a proportion (*P*) of 50% was assumed, with a 95% confidence level (*Z* = 1.96) and a precision (*ε*) of 5%. This yielded a minimum sample size of 385. Considering a 10% nonresponse rate, the final sample size was adjusted to 427.

Multiple sampling strategies were employed to select study participants. First, a stratified sampling technique was used, dividing pharmacies in Dar es Salaam into strata based on their location within the five municipalities: Kinondoni, Ilala, Temeke, Ubungo, and Kigamboni. Next, to ensure proportional representation, the sample size for each municipality was determined by calculating the proportion of pharmacies in each municipality relative to the total number of pharmacies in Dar es Salaam. This proportion was then multiplied by the required sample size, resulting in 132 pharmacies in Kinondoni, 150 in Ilala, 63 in Ubungo, 61 in Temeke, and 21 in Kigamboni.

Within each municipality, community pharmacies were selected using a simple random sampling technique. Only one dispenser was interviewed per selected pharmacy.

### 2.4. Data Collection Procedures

A structured, modified questionnaire, adapted from a study conducted in Senegal [29], was used to collect data from study participants. The questionnaire comprised the following four sections:

• Section A collected sociodemographic information, including sex, marital status, educational level, and profession.
• Section B listed six BZPs, and respondents were asked to rank them based on dispensing frequency.
• Section C assessed knowledge of the pharmacological effects of BZPs (4 variables) and their side effects or associated risks (12 variables).
• Section D contained 14 variables evaluating dispensing practices of BZPs, categorized into three areas: verification of prescription authenticity (8 variables), advising clients on the risks of BZP misuse (3 variables), and the dispenser's decision on nonprescription requests for BZPs (3 variables).

Section C and D questions required yes or no responses. Data were collected through face-to-face interviews conducted by trained final-year Bachelor of Pharmacy students after obtaining written informed consent from the community pharmacy dispensers.

Overall knowledge and practice were assessed by calculating the mean scores for the respective questions, with correct responses assigned a score of 1 and incorrect responses a score of 0. Skewness was tested, and the scores were found to be normally distributed. Participants were categorized based on the mean score: those scoring at or above the mean were classified as having adequate knowledge (mean = *X*) or good practice (mean = *Y*), while those scoring below the mean were classified as lacking adequate knowledge or good practice.

### 2.5. Data Analysis

Data were entered, cleaned, and analyzed using SPSS Version 23. The results are presented as frequencies, means, and percentages. Relationships between variables were assessed using Pearson's chi-square test.

Stepwise binary logistic regression was performed to identify factors influencing BZP-dispensing practices. Variables with a *p* value of ≤ 0.2 in Pearson's chi-square test were included in the univariate analysis, and those meeting the same threshold were further subjected to multivariate analysis. Statistical significance was set at a *p* value of < 0.05.

## 3. Results

### 3.1. Sociodemographic Characteristics of the Participants

Out of the 427 dispensers visited, 378 consented to participate in the study (response rate = 88%). The main reasons for not consenting were not being willing to participate. Of the 378 participants, 232 (61.4%) were female, 263 (69.6%) had a college education level, and 193 (51.1%) were pharmaceutical technicians (Table 1).

### 3.2. Most Dispensed BZP

**Figure 1:**
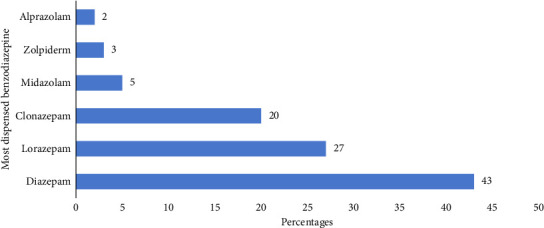
The most dispensed benzodiazepines.

Diazepam was the most (163 (43%)) dispensed BZPs, followed by lorazepam (102 (27%)), clonazepam (76 (20%)), and midazolam (18 (5%)) (Figure 1).

### 3.3. Community Pharmacy Dispenser's Knowledge of BZPs

The majority (304 (80.4%)) of the study participants knew sedation as one of the pharmacological effects of BZPs, followed by anticonvulsant (153 (40.5%)), myorelaxation (130 (34.4%)), and anxiolysis (109 (28.8%)). Besides, the majority knew drowsiness (343 (90.7%)), behavioral disorder (205 (54.2%)), and impaired psychomotor function (201 (53.2%)) as BZP side-effects. Overall, 175 (46.3%) had adequate knowledge of BZP pharmacological indications and common side effects, Table 2.

### 3.4. Dispensing Practices of BZPs Among Participants

Most of the participants acknowledged that they verified key BZP prescription information before dispensing, namely, prescriber's stamp (268 (70.9%)), patient's name (334 (88.4%)), name of the prescriber (320 (84.7%)), dosage (376 (99.5%)), and prescription date (249 (65.9%)). On advising clients on the risks of misuse of BZPs, half of the dispensers (193 (51.1%)) advise consumers/patients at each visit. Overall, 240 (63.5%) of the dispensers had good BZP dispensing practices (Table 3).

### 3.5. Determinants of Good Dispensing Practice of BZPs

Sex, marital status, and educational level were not associated with good dispensing practices of BZPs. In multivariable analysis, being a nonpharmaceutical professional was 6.25 less likely to have good dispensing practice than pharmacists (AOR = 0.16, 95% CI (0.05–0.49)) and having adequate knowledge of BZPs was 2.6 more likely to have good dispensing practice than those with inadequate knowledge (AOR = 2.64, 95% CI (1.64–4.25)), and the differences were statistically significant, Table 4.

## 4. Discussion

This study aimed to assess the determinants of BZP-dispensing practice among community pharmacy dispensers in Dar es Salaam, Tanzania. The study found that diazepam was the most (163 (43%)) dispensed BZPs. Among the study respondents, 175 (46.3%) had adequate knowledge of the pharmacological effects and side effects of BZPs and 240 (63.5%) of community dispensers had good dispensing practices. Besides, determinants of the good dispensing practice of BZPs were found to be the level of professional education and knowledge of BZPs.

Of the six BZPs in this study, diazepam was the most dispensed; findings are similar with studies from Spain, Brazil, Senegal, and South Africa [6, 13, 29, 38]. This trend is attributed to its wide range of uses in psychiatric, neurocognitive, and chronic pain conditions, its long half-life, and affordability [13, 20]. Literature highlights that diazepam is widely available and popular among community pharmacy dispensers in LMICs [12, 13, 20, 39]. A study conducted in Tanzania in 2019 further reported that 70% of community pharmacy dispensers were willing to dispense diazepam without a prescription [20]. These findings underscore the need for stricter regulations, guidelines, and monitoring to curb potential misuse.

Our study revealed that only 46.3% of the dispensers had adequate knowledge of the pharmacological uses and potential side effects of BZPs. However, the majority were aware that BZPs are used for sedation (80.4%) and can cause drowsiness (90.7%). These findings align with studies conducted in Senegal and Ethiopia, which also reported low levels of knowledge among community pharmacy dispensers [29, 32]. The observed knowledge gap is likely linked to the common practice in LMICs of hiring low-cadre or unqualified personnel in community pharmacies [12]. In our study, only 13.5% of the dispensers were pharmacists while 15.3% were nonpharmaceutical personnel. In addition, the absence of on-the-job learning platforms hampers the continuous improvement of knowledge and skills among dispensers. This is supported by a study conducted by Shankar et al., which emphasized that continuing professional education is critical for enhancing and maintaining the knowledge of community pharmacy dispensers [40].

The study also found that approximately 63.5% of community pharmacy dispensers demonstrated good dispensing practices for BZPs. This was primarily attributed to their practice of verifying the authenticity of prescriptions. However, the majority of dispensers exhibited poor practices in key areas, including advising clients on the risks of BZP misuse (> 60%) and dispensing BZPs without a prescription (96.6%). These findings align with a study conducted in Pakistan, where only half of the community pharmacists consistently educated clients about adhering to their medications [12]. Similarly, in China, just 17.5% of community pharmacists provided proper counseling on medications [41]. The high willingness to dispense BZPs without prescriptions observed in our study is consistent with previous research conducted in Tanzania and Ethiopia [20, 32]. The poor dispensing practices identified in this study may be attributed to weak regulatory enforcement in many LMICs [35, 42]. This highlights the need for stricter oversight and improved guidelines to ensure the responsible dispensing of BZPs.

Lastly, educational level, professional training, and overall knowledge were identified as statistically significant determinants of good BZP dispensing practices. Consistent with our findings, a study by Tine et al. [29] reported a significant association between education level and improved dispensing practices among medicine dispensers. However, Mekonnen and Beyna found no significant link between dispenser knowledge and dispensing practices [32]. This discrepancy may stem from differences in the economic and regulatory environments of the study settings. In developed countries, dispensers are typically more knowledgeable due to higher standards of education and training, and regulatory authorities strictly enforce laws and guidelines [43]. Our findings emphasize the critical need to employ individuals with adequate pharmaceutical knowledge and skills in community pharmacies to promote the rational use of medications. This approach would enhance the quality of dispensing practices and contribute to better healthcare outcomes.

The primary limitation of this study was the absence of direct observation of BZP-dispensing practices among community dispensers, which may have resulted in an overestimation of good practices. Consequently, the findings should be interpreted with caution. To address potential recall bias, the study employed a carefully structured questionnaire with a logical sequence of questions designed to accurately assess participants' knowledge and practices. Moreover, we encountered a higher rate (12%) than the anticipated nonresponse rate (10%), which affected the precision of the estimates.

## 5. Conclusion

The level of knowledge and the qualifications of pharmaceutical professionals are critical determinants of good BZP-dispensing practices among community pharmacy dispensers. Employing unqualified pharmaceutical personnel increases the potential risks to consumers of BZPs. Therefore, strict enforcement of regulations governing the registration of community pharmacy dispensers is essential, along with regular training to reduce the misuse of BZPs. Further research is needed to evaluate the availability and effectiveness of training and continuing professional education programs for community pharmacy dispensers.

## Figures and Tables

**Table 1:** Sociodemographic characteristics of the participants, *n* = 378.

Variable	*n* (%)
Sex	
Male	146 (38.6)
Female	232 (61.4)
Marital status	
Unmarried	242 (64)
Married	136 (36)
Education level	
Secondary level	36 (9.5)
College level (diploma and certificate levels)	263 (69.6)
University level (degree and above)	79 (20.9)
Profession	
Pharmacists	51 (13.5)
Pharmaceutical technician	193 (51.1)
Pharmaceutical assistance	21 (5.6)
Pharmaceutical dispensers	17 (4.5)
Accredited drug dispensing outlets (ADDO) certificate holders	38 (10.1)
Nonpharmaceutical personnel	58 (15.3)

**Table 2:** Knowledge of benzodiazepines among pharmacy dispensers.

Knowledge items	Response *n* (%)	
	Yes	No
Pharmacological indications of benzodiazepines		
Sedative	304 (80.4)	74 (19.6)
Anxiolytic	109 (28.8)	269 (71.2)
Anticonvulsant	153 (40.5)	225 (59.5)
Muscle relaxant	130 (34.4)	248 (65.6)
Common side-effects of benzodiazepines		
Drowsiness	343 (90.7)	35 (9.3)
Behavioral disorder	205 (54.2)	173 (45.8)
Confusion	186 (49.2)	192 (50.8)
Libido disorders	81 (21.4)	297 (78.6)
Hypo vigilance	93 (24.6)	285 (75.4)
Impaired psychomotor function	201 (53.2)	177 (46.8)
Agitation	91 (24.1)	287 (75.9)
Aggressiveness	96 (25.4)	282 (74.6)
Irritability	157 (41.5)	221 (58.5)
Anterograde amnesia	65 (17.2)	313 (82.8)
Tolerance	162 (42.9)	216 (57.1)
Addiction	270 (71.4)	108 (28.6)
Overall level of knowledge of benzodiazepines		
Adequate	175 (46.3)	
Inadequate	203 (53.7)	

**Table 3:** Dispensing practices of benzodiazepines among community pharmacy dispensers.

Dispenser's practice	Response *n* (%)	
	Yes	No
Verification of elements of the authenticity of the prescription		
Prescriber's stamp	268 (70.9)	110 (29.1)
Patient's name	334 (88.4)	44 (11.6)
Name of the prescriber	320 (84.7)	58 (15.3)
Dosage	376 (99.5)	2 (0.5)
Prescription date	249 (65.9)	129 (34.1)
Duration of use	365 (96.6)	13 (3.4)
Address of the prescriber	277 (73.3)	101 (26.7)
Advising clients on the risks of misuse of BZDs		
On request	101 (26.7)	277 (73.3)
At each visit	193 (51.1)	185 (48.9)
Only if necessary	134 (35.4)	244 (64.6)
Pharmacy provider's decision on a nonprescription request for BZDs		
Acceptance of delivery	115 (30.4)	263 (69.6)
Refusal to issue	9 (3.4)	263 (96.6)
Registration of the prescription	176 (46.6)	202 (53.4)
Overall practice of benzodiazepines		
Good	240 (63.5)	
Poor	138 (36.5)	

**Table 4:** Determinants of good dispensing practice of benzodiazepine, *n* = 240.

Variable	Good *n* (%)	*P* value	Univariate	Multivariate
			COR (95% CI)	AOR (95% CI)
Sex				
Male	88 (60.3)	0.324		
Female	152 (65.5)			
Marital status				
Unmarried	161 (66.5)	0.119	Ref	Ref
Married	79 (58.1)		0.69 (0.45–1.07)	0.91 (0.55–1.50)
Educational level				
Secondary	17 (47.2)	0.002	Ref	Ref
Collage	182 (69.2)		2.51 (1.24–5.08)	2.40 (0.65–8.80)
University	41 (51.9)		1.20 (0.54–2.65)	2.04 (0.42–9.90)
Pharmaceutical professional				
Pharmacist	35 (68.6)	≤ 0.001	Ref	Ref
Pharmaceutical technician	141 (73.1)		9.34 (3.86–22.61)	1.23 (0.29–5.25)
Pharmaceutical assistant	15 (71.4)		11.58 (5.58–24.02)	1.28 (0.22–7.29)
Pharmaceutical dispenser	12 (70.6)		10.68 (3.37–33.80)	1.20 (0.21–6.67)
ADDO holder	26 (68.4)		10.25 (2.98–35.17)	1.84 (0.35–9.70)
Nonpharmaceutical personnel	11 (19)		9.25 (3.58–23.89)	0.16 (0.05–0.49)
Overall knowledge of BZDs				
Inadequate	89 (50.9)	≤ 0.001	Ref	Ref
Adequate	151 (74.4)		2.80 (1.82–4.32)	2.64 (1.64–4.25)

## Data Availability

The data that support the findings of this study are available on request from the corresponding author. The data are not publicly available due to privacy or ethical restrictions.
